# Effects of Water Content and Particle Size on Yield and Reactivity of Lignite Chars Derived from Pyrolysis and Gasification

**DOI:** 10.3390/molecules23102717

**Published:** 2018-10-22

**Authors:** Yong Huang, Yonggang Wang, Hao Zhou, Yaxuan Gao, Deliang Xu, Lei Bai, Shu Zhang

**Affiliations:** 1 College of Materials Science and Engineering, Nanjing Forestry University, Nanjing 210037, China; huangyonghn@163.com (Y.H.); spraaa@126.com (H.Z.); 18260077429m@sina.cn (Y.G.); xudl@njfu.edu.cn (D.X.); 2 School of Chemical and Environmental Engineering, China University of Mining and Technology (Beijing); Beijing 100083, China; yg_wang1@sina.com; 3 Department of Chemical and Biomedical Engineering, West Virginia University, Morgantown, WV 26506, USA

**Keywords:** pyrolysis, gasification, water content, char reactivity, lignite

## Abstract

Water inside coal particles could potentially enhance the interior char–steam reactions during pyrolysis and gasification. This study aims to examine the effects of water contents on the char conversion during the pyrolysis and gasification of Shengli lignite. The ex-situ reactivities of chars were further analyzed by a thermo gravimetric analyzer (TGA). Under the pyrolysis condition, the increase in water contents has monotonically decreased the char yields only when the coal particles were small (<75 µm). In contrast, the water in only large coal particles (0.9–2.0 mm) has clearly favored the increase in char conversion during the gasification condition where 50% steam in argon was used as external reaction atmosphere. The waved reactivity curves for the subsequent char–air reactions were resulted from the nature of heterogeneity of char structure. Compared to the large particles, the less interior char–steam reactions for the small particles have created more differential char structure which showed two different stages when reacting with air at the low temperature in TGA.

## 1. Introduction

With the continual diminishing of high-rank coal, lignite as a low-rank fuel is becoming an increasingly important resource for producing chemicals and generating energy [1,2,3,4]. The high-water content which sometimes exceeds 50 wt % for some lignites greatly restricts its utilization. Therefore, dewatering is the first and essential step in almost all lignite utilization processes. A number of methods for dewatering lignite, such as flue gas drying in fluidized bed [5], tube type drying technology [6], hydrothermal dewatering [7,8,9], and mechanical/thermal dewatering [10,11,12,13], etc., have been tried and developed. However, dewatering coal on a large scale is always very energy-intensive, especially for those processes requiring a very deep extent of drying. It will greatly favor the economic feasibility if (partially dried) lignite containing a certain level of water could be used as feedstock. 

The tolerance of a technology process to water content in lignite could be vastly different. For instance, the fast pyrolysis for producing bio-oil must have a coal sample with minimal water. Any water remaining in the feedstock will be transferred to the oil product during the condensation stage, increasing the difficulty in the subsequent upgrading treatment and dramatically degrading the oil value. Recently, a novel Oxy–Steam Combustion System was proposed and developed [14,15], which could likely improve the efficiency of heat transfer by the direct contact between steam and the combustion zone. A certain moisture in coal samples for the O_2_/steam combustion system could not see any foreseeable negative impacts in terms of the reaction itself. Indeed, it may reduce the requirement of external input of water/steam, thus, decreasing the operation cost. Similarly, the gasification system may not have a critical demand on the water content for the feedstock as steam is always one of the key gasifying agents. Actually, the inherent water in the coal could possibly be conducive to improving gasification efficiency. In the high-temperature environment in a gasifier, the water in the coal will evaporate and turn into steam inside coal particles. The steam can immediately react with adjoining carbon in the internal particles of coal or char without the need to wait for the external steam to diffuse into the particles. In other words, the gasification rate could potentially be enhanced due to the availability of steam inside the char particles. Moreover, the different forms of water (e.g., free water, chemically-absorbed water, etc.) [16,17,18,19,20] in coal means that the water molecules will gradually travel out of the coal particles, giving a strong chance for the char–steam reactions in the pore channels to occur. 

The information about the effect of water content on coal conversion is lacking in the literature so far. It is, therefore, important to examine the role of inherent water inside particles on the coal gasification behavior, particularly for lignite that features a high reactivity and, thus, increases the likelihood of carbon–steam reactions inside particles. The purpose of the present work is to examine the dependence of char conversion and reactivity on the inherent water during the pyrolysis and steam gasification using a Chinese Shengli lignite with two particle sizes. The pyrolysis condition provides a case to investigate the changes in char conversion/reactivity in the absence of an external steam supply. The results from this study indicate that the interior char–steam reactions were considerably affected by the particle size and the availability of external steam.

## 2. Results and Discussion

### 2.1. Effect of Water Content on Char Yield

**Figure 1:**
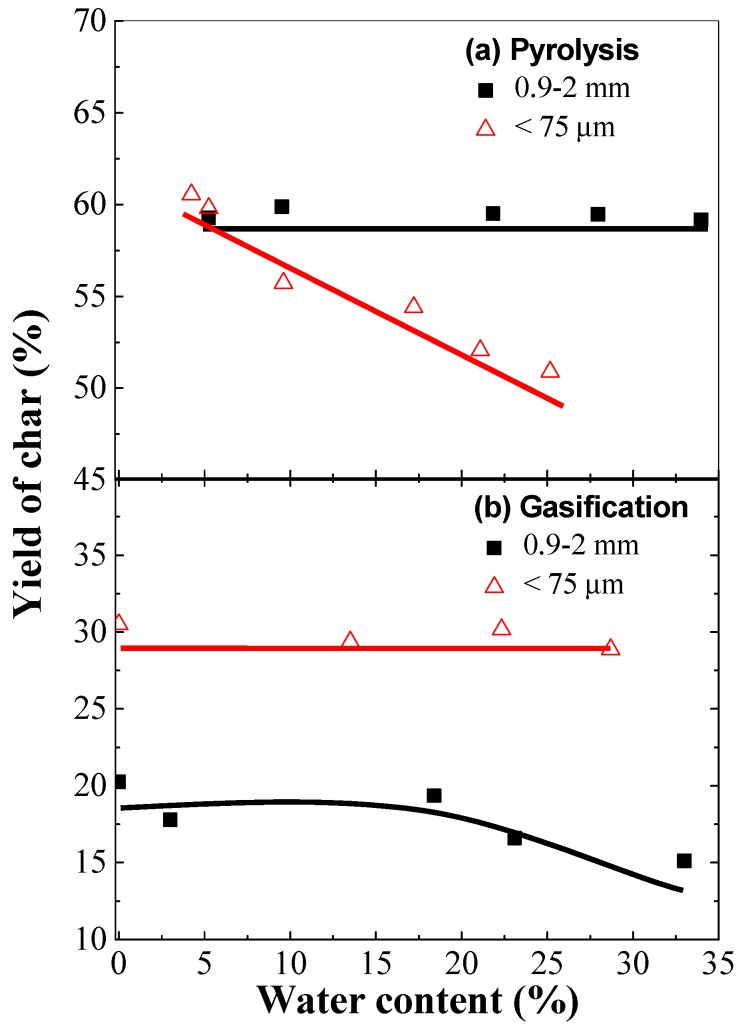
Char yields as a function of water contents in coals with different particle sizes in the absence (**a**) and presence (**b**) of steam as the reaction atmosphere respectively.

Figure 1 illustrates the effect of water contents on char yields during the pyrolysis (a) and gasification (b) of lignite with two different coal particle sizes at 900 °C in the fixed bed reactor. Clearly, it can be seen from the comparison of Figure 1a,b that the char yields from pyrolysis were obviously higher than those from the gasification in steam. In the absence of steam in Figure 1a, the influence of inherent water on char yields is negligible for the big coal particles (0.9–2 mm) while the char conversion from the small coal particles (<0.075 mm) monotonically increases with the increase in the water content in coal. Comparatively, in the presence of steam in Figure 1b, it is interesting to note that the effect of water content on char yield is very little in the case of small coal particles, but a decrease in char yield was clearly observed with a rise of water content in the large coal particles.

For the large coal particles, the heat transfer from outer to inner particles is slow owing to the nature of weak heat conductivity of char, leading to the slow heating rate inside the coal particles. Thus, the time taken to reach steam gasification temperatures (>700 °C) inside the large char particles has allowed the inherent water to evaporate and travel out of inner particles. The absence of char–steam reactions inside the particles is the key to understand the almost constant char yield in Figure 1a. However, the presence of steam in the reaction atmosphere (steam gasification condition) surrounding the char particles could inhibit the internal steam diffusion, and then the residence time of steam in the particle could be extended. This provides a chance for interior char–steam reactions to occur when the gasification temperature is reached, which is the main reason for the decrease in char yield with an increase in water content for the large coal particles, as shown in Figure 1b. 

For the small coal particles, the char yield decreases with a rise of water content during pyrolysis as shown in Figure 1a, whereas the inherent water seems to have a little effect on the char yield during the steam gasification as shown in Figure 1b. The heat can immediately transfer into the inner area of small coal particles to reach the gasification temperature as soon as the coal is fed into the reactor. When the small coal particles with different water content are gasified in the atmosphere of steam (50 vol %), the low diffusion resistance from the exterior to interior particles results in a lower effect of inherent water on the char conversion.

Another abnormal phenomenon is that the char yields from large coal particles are generally lower than those from the small coal particles in the case of gasification in steam, as shown in Figure 1b. As mentioned before, some water was associated with O-containing functional groups inside the lignite particles. At high temperatures, the co-existence of steam and highly-active O-containing radicals may easily activate the interior char and, thus, facilitate char–steam reactions. Additionally, the absolute “0%” water content could not be guaranteed as there was a short period when the dried sample was exposed to air during loading to the feeder. However, the experimental errors would exert little effect on the trends and conclusions obtained from this work. 

### 2.2. Effect of Water Content on Ex-Situ Char Reactivity

**Figure 2:**
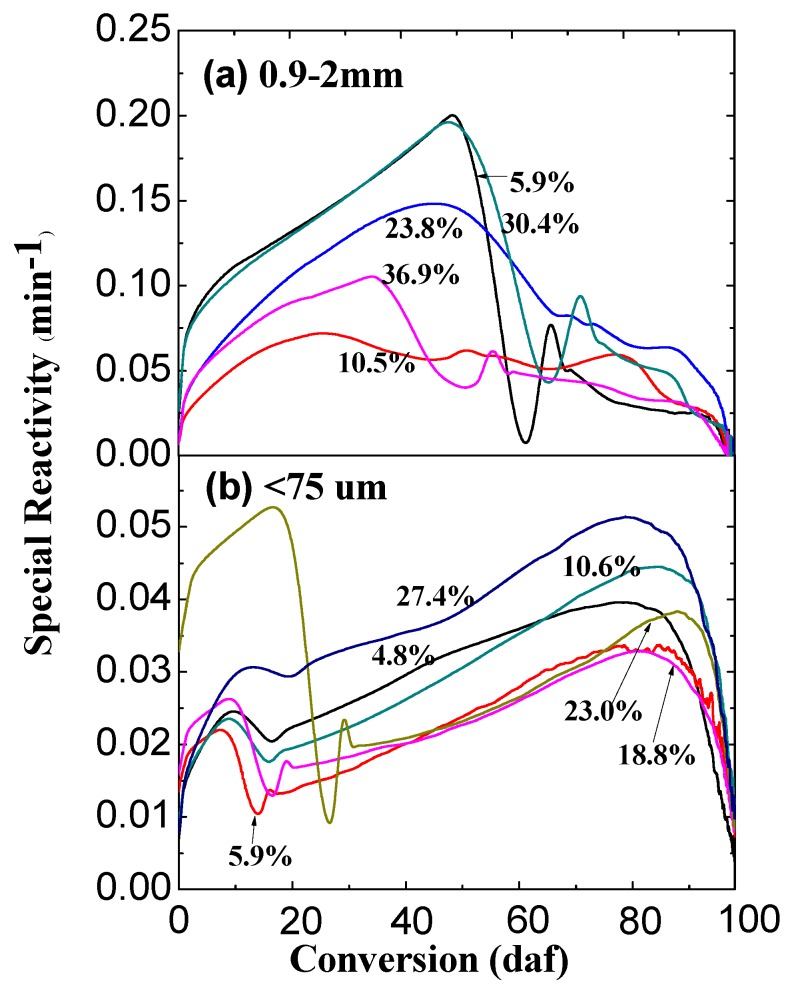
Char reactivity in air at 400 °C as a function of char conversion level. The chars were prepared from the pyrolysis of Shengli lignite samples with different water content at 900 °C (The numbers on curves refer to the water content of the coal samples). Different coal particle sizes for char preparation were 0.9–2 mm (**a**) and <0.075 mm (**b**) as labeled in the graphs, respectively.

Figure 2 shows the specific reactivities of chars prepared from the pyrolysis of Shengli lignite with different water content in nitrogen in the fixed-bed reactor at 900 °C. The reactivities of char from large particle size coals are higher than those of small ones (the y-axis scales are different between Figure 2a,b). Compared to the poor interior char–steam reactions in the large particles, both interior and exterior steam–char reactions in the small particles have together consumed the char matrix significantly, leaving the less reactive char as shown in Figure 2b. However, the char reactivity does not show a clear increasing trend with the increase in water content. Heterogeneity of char structures means that the char–steam reactions and char–radical interactions could be somehow complex and selective, thus, altering the subsequent char–air reactivity in an unpredictable way. For example, the char conversions for the large particles shown in Figure 1 are nearly the same, but with very different char–air reactivities as shown in Figure 2a.

**Figure 3:**
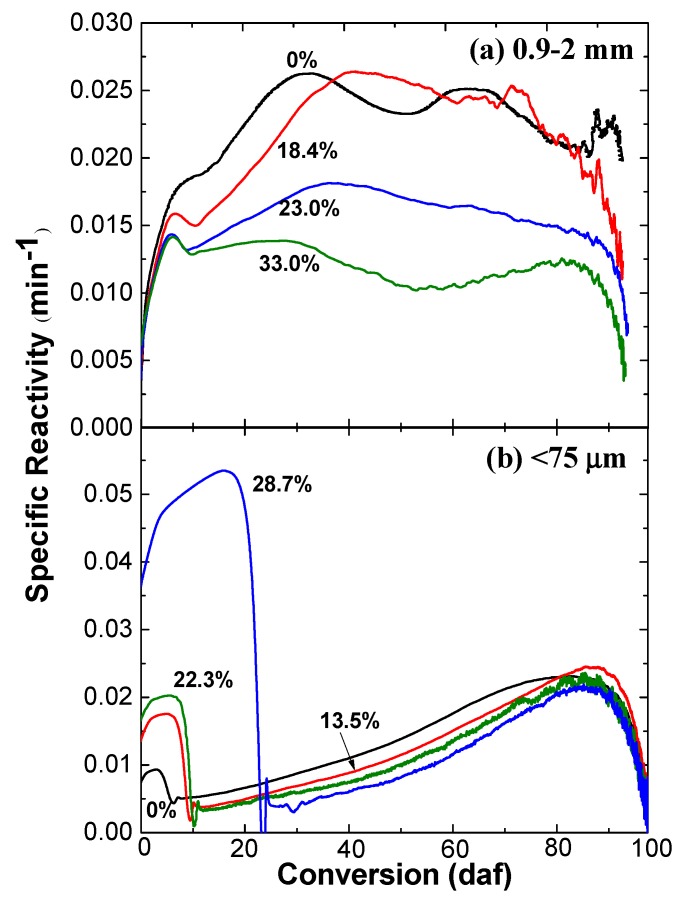
Char reactivity in air at 400 °C as a function of char carbon conversion level. The chars were prepared from the gasification of Shengli lignite samples with different inherent water content at 900 °C in steam and nitrogen (the numbers on curves refer to the inherent water content of the coal sample). Different coal particle sizes for char preparation were 0.9–2 mm (**a**) and <0.075 mm (**b**) as labeled in the graphs, respectively.

Figure 3 shows the specific reactivities of chars from the gasification in steam of coals with different water content. For the large particles, the char reactivity decreases with an increase in the water content. As mentioned above, the water could exist inside the coal particles for a relatively long time due to the presence of steam in the reaction zone. The interior char–steam reactions could preferentially consume the active carbon structure as well as initiate/enhance the ring condensation reactions [21,22,23], leading to a lower char reactivity.

For the small particles, the changes in the reactivities of char–air reactions are clearly divided into two stages, as shown in Figure 3b. In the first stage, the increase in water content leads to a rise of the char–air reaction reactivity, while the reactivity clearly decreases with the increase in water content at the second stage. Based on the above discussion, the extent of interior char–steam reactions in the large particles is much more significant, compared to that in the small particles under the gasification condition. Figure 3a,b seems to suggest that the interior char–steam reactions and exterior char–steam reactions have exerted completely different impacts on the subsequent char–air reactions. It is speculated that the balance between interior and exterior char–steam reactions in the case of large particles is favorable to form char with relatively uniform char structure and, thus, less waved char–air reactivity. In contrast, the less significant interior char–steam reactions in the case of small particles create more heterogeneous char structures which show two different stages when reacting with air at the low temperature in TGA. 

## 3. Materials and Methods

### 3.1. Coal Sample 

A Shengli lignite from China was used as the raw material in this study. The as-received coal was crushed and sieved to obtain two fractions one with a large particle size (0.9–2 mm) and the other with a small particle size (less than 0.075 mm) for carrying out the experiments. The two fractions were dried in air at room temperature with different holding periods to acquire coal samples with varied water content as shown in Table 1. The properties of the Shengli lignite are C, 64.39; H, 4.50; N, 1.21; S, 0.42; O, 29.48, and volatile matter, 46.26 (wt %, daf) with an ash yield of 7.99 (wt %, db).

### 3.2. Pyrolysis and Gasification

**Figure 4:**
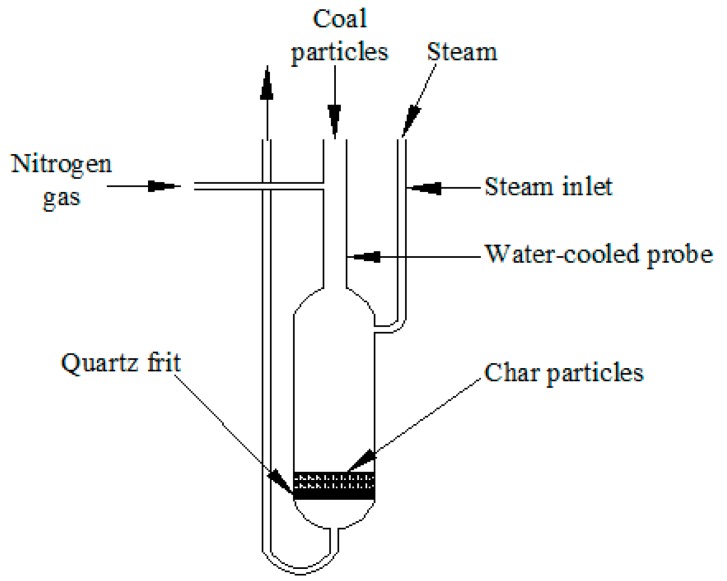
A schematic diagram of the fixed bed reactor used in this study.

The experiments were classified into two types according to the reaction atmospheres. In the first type of experiment (pyrolysis), 1.25 g coal samples with different water content were dropped directly into the reactor within seconds under a continuous flow of nitrogen at 0.75 L/min. Before feeding, the reactor was heated and maintained at 900 °C. The sample was then held for 10 min in the nitrogen atmosphere at the target temperature. The second type of experiment (gasification) was conducted in 50 vol % steam as the reaction atmosphere with the same total flow rate of 0.75 L/min. Steam was generated by feeding water directly into the reactor with a peristaltic pump (LongerPump BT100-1F, Baoding, China). The char yields were obtained by weighing the reactor shown in Figure 4 before and after experiments. The reaction zone in the quartz reactor was 40 mm in diameter and 130 mm in height.

### 3.3. Reactivity Measurement 

The Perkin-Elmer Pyris 1 thermo gravimetric analyzer (TGA) (Perkin-Elmer, Walther, MA, USA) with a high temperature furnace was used for measuring the reactivity of the chars after pyrolysis and gasification in the fixed-bed reactor (Daxingkete, Beijing, China). The chars were stored in sealed bottles in a freezer before being subjected to the reactivity measurement in TGA. About 7 mg of char was placed in a Pt crucible and heated in nitrogen to 105 °C and was held at that temperature for 20 min to remove the water from the char. The stabilized weight of char at 105 °C was taken as the weight of dry char. The char was then heated in nitrogen to 400 °C. The isothermal reactivity measurement commenced when the gas atmosphere was switched from nitrogen to air. A temperature of 400 °C as the reaction temperature was chosen in this study to minimize the changes in char structure due to thermal annealing [21,22,24]. The specific reactivity (R) of char at any given time was calculated from the DTG (derivative thermogravimetric analysis) using the software (Perkin-Elmer, Pyris 11.0, Walther, MA, USA) data (dW/dt).

$$R=-\frac{1}{W}\frac{\mathrm{dW}}{\mathrm{dt}}$$

where W is the weight (daf basis) of the char at any given time t.

## 4. Conclusions

This study has indicated that the water content in lignite could not only affect the char conversions during the pyrolysis and gasification in steam, but also impact the subsequent char–air reactions. Under the pyrolysis condition, the char conversion decreases with the increase in water content in the case of the small particles, while the interior char–steam clearly increases with the rise of water content for the large particles in the gasification condition. The interior char–steam reactions cause the lower char yield for the large particles than that for the small particles in the gasification condition. The heterogeneity of char after experiencing the char–steam reactions leads to the waved reactivity curves from the subsequent char–air reactions in TGA. Compared to the large particles, the less significant interior char–steam reactions in the case of small particles create more heterogeneous char structures which show dual-stage reactivity curves when reacting with air at a low temperature in TGA. 

## Figures and Tables

**Table 1:** Water contents of coal samples.

	Pyrolysis		Gasification	
	0.9–2 mm	<75 µm	0.9–2 mm	<75 µm
Water Content (wt %)	5.9	4.8	0	0
	10.5	5.9	18.4	13.5
	23.8	10.6	23.0	22.3
	30.4	18.8	33.0	28.7
	36.9	23.0		
		27.4		
